# Conservation and diversity of the IrrE/DdrO‐controlled radiation response in radiation‐resistant *Deinococcus* bacteria

**DOI:** 10.1002/mbo3.477

**Published:** 2017-04-11

**Authors:** Laurence Blanchard, Philippe Guérin, David Roche, Stéphane Cruveiller, David Pignol, David Vallenet, Jean Armengaud, Arjan de Groot

**Affiliations:** ^1^ Lab Bioenerget Cellulaire CEA, DRF, BIAM Saint‐Paul‐lez‐Durance France; ^2^ CNRS UMR 7265 Biol Veget & Microbiol Environ Saint‐Paul‐lez‐Durance France; ^3^ Aix‐Marseille Université Saint‐Paul‐lez‐Durance France; ^4^ Laboratory “Innovative technologies for Detection and Diagnostic” CEA‐Marcoule DRF/IBITEC‐S/SPI/Li2D Bagnols‐sur‐Cèze France; ^5^ CEA, DRF Institut de Génomique LABGeM Evry France; ^6^ UMR‐CNRS 8030 Génomique Métabolique CEA Institut de Génomique – Genoscope Evry France

**Keywords:** gene regulation, proteomics, regulon, transcriptional repressor, transcriptomics, zinc peptidase

## Abstract

The extreme radiation resistance of *Deinococcus* bacteria requires the radiation‐stimulated cleavage of protein DdrO by a specific metalloprotease called IrrE. DdrO is the repressor of a predicted radiation/desiccation response (RDR) regulon, composed of radiation‐induced genes having a conserved DNA motif (RDRM) in their promoter regions. Here, we showed that addition of zinc ions to purified apo‐IrrE, and short exposure of *Deinococcus* cells to zinc ions, resulted in cleavage of DdrO in vitro and in vivo, respectively. Binding of IrrE to RDRM‐containing DNA or interaction of IrrE with DNA‐bound DdrO was not observed. The data are in line with IrrE being a zinc peptidase, and indicate that increased zinc availability, caused by oxidative stress, triggers the in vivo cleavage of DdrO unbound to DNA. Transcriptomics and proteomics of *Deinococcus deserti* confirmed the IrrE‐dependent regulation of predicted RDR regulon genes and also revealed additional members of this regulon. Comparative analysis showed that the RDR regulon is largely well conserved in *Deinococcus* species, but also showed diversity in the regulon composition. Notably, several RDR genes with an important role in radiation resistance in *Deinococcus radiodurans*, for example *pprA*, are not conserved in some other radiation‐resistant *Deinococcus* species.

## 1 Introduction

Exposure to high doses of ionizing radiation is lethal for most known living organisms. *Deinococcus* bacteria, however, are extremely tolerant to gamma and UV radiation as well as to desiccation and other DNA damage‐ and oxidative stress‐generating conditions. The underlying mechanisms are not fully understood, but several factors that are crucial for this extraordinary tolerance have been described, including limitation of oxidative protein damage, repair of massive DNA damage and an efficient SOS‐independent damage response pathway (Confalonieri & Sommer, 2011; Cox & Battista, 2005; Daly, 2012; Ludanyi et al., 2014; Slade & Radman, 2011).

Transcriptome analyses of *Deinococcus radiodurans* (Liu et al., 2003; Tanaka et al., 2004) and *Deinococcus deserti* (de Groot et al., 2014) after exposure to gamma radiation showed induced expression of many genes, including several required for DNA repair (e.g., *recA*). Several highly induced novel genes were also identified and designated *ddrA* to *ddrP* and *pprA*, and the contribution to radiation tolerance in *D. radiodurans* was demonstrated for *pprA* and *ddrA* to *ddrD* (Tanaka et al., 2004). In most characterized bacterial species, expression of *recA* and other DNA repair genes is controlled by LexA, the repressor of the well‐known SOS reponse, but LexA is irrelevant to *recA* induction in *D. radiodurans* (Narumi et al., 2001; Sheng, Zheng, Tian, Shen, & Hua, 2004). Characterization of radiation‐sensitive *D. radiodurans* mutant strains led to the identification of another novel gene, the constitutively expressed *irrE*, which was shown to be required, at the time in an unknown manner, for induced expression of *recA* and *pprA* following exposure to radiation (Earl, Mohundro, Mian, & Battista, 2002; Hua et al., 2003). A conserved 17‐base pair palindromic sequence, designated radiation/desiccation response motif (RDRM), was found at a variable position upstream of about 20 radiation‐induced genes in *D. radiodurans*, including *recA* and *pprA*, supporting the existence of a radiation/desiccation response (RDR) regulon (Makarova et al., 2007). Although IrrE was shown to be necessary for induction of *recA* and *pprA*, another protein, the helix‐turn‐helix XRE‐family protein DdrO, was proposed to be the transcriptional regulator for the RDR regulon because *ddrO* itself, unlike *irrE*, is radiation‐induced and preceded by an RDRM site (Makarova et al., 2007).

IrrE, DdrO, and the RDRM are highly conserved in *Deinococcus* species, and the predicted RDR regulons of *D. radiodurans*,* D. deserti*, and *Deinococcus geothermalis* have 16 genes in common (*ddrB*,* ddrC*,* ddrD*,* ddrO*,* pprA*,* ssb*,* gyrA*,* gyrB*,* recQ*,* uvrA*,* uvrB*,* uvrD*,* tkt*, operon *cinA*‐*ligT*‐*recA*) but also contain several genes that are not found in each species (e.g., *Deide_02842* encoding a restriction enzyme, and *Deide_04721* and the five‐gene operon *Deide_18730* to *Deide_18690* of unknown function in *D. deserti*) (de Groot et al., 2009, 2014; Ludanyi et al., 2014; Makarova & Daly, 2010; Makarova et al., 2007). Moreover, *D. deserti* contains a second *ddrO* and also two additional *recA* genes, each of which is radiation‐induced and preceded by an RDRM (de Groot et al., 2014; Ludanyi et al., 2014). Like in *D. radiodurans*, IrrE of *D. deserti* was shown to be required for radiation resistance and for the radiation‐induced expression of at least three genes of the RDR regulon (i.e., its three *recA* genes that code for two functionally different RecA proteins called RecA_C_ and RecA_P_) (Dulermo, Fochesato, Blanchard, & de Groot, 2009; Vujicic‐Zagar et al., 2009).

Analysis of the IrrE sequence revealed the presence of the conserved domain COG2856 (predicted Zn peptidase), which contains the HEXXH motif suggestive of a zinc‐binding catalytic active site. The crystal structure of *D. deserti* IrrE has been solved, which showed structural similarity of its N‐terminal domain with thermolysin, a zinc metalloenzyme of *Bacillus thermoproteolyticus* (Vujicic‐Zagar et al., 2009). A zinc ion was found in the predicted zinc‐binding site after soaking apo‐IrrE crystals for two minutes in a solution containing ZnSO_4_. *Deinococcus deserti* strains expressing IrrE with mutations E83Q or H86S in the HEXXH motif were as sensitive to radiation as a *D. deserti irrE* deletion mutant. These data suggested that IrrE could be a zinc peptidase (Vujicic‐Zagar et al., 2009).

Insight into the regulatory mechanism leading to radiation‐induced expression of *recA* and other genes in *Deinococcus* was obtained only recently. First, following the genome‐wide identification of transcription start site (TSS) positions in *D. deserti* it was found that the RDRM sites are located in or very close to the promoters of radiation‐induced genes, indicating that an RDRM‐binding protein would function as a repressor (de Groot et al., 2014). Second, studies on *D. deserti* provided evidence for a novel radiation response mechanism that involves both IrrE and DdrO in the transcriptional control of the RDR regulon (Ludanyi et al., 2014). It was demonstrated that IrrE is indeed a metalloprotease and that it cleaves and inactivates DdrO. Uncleaved DdrO was shown to form at least dimers and to function as a repressor of an RDRM‐containing promoter. Cleavage of the RDR repressor DdrO by a separate protease is thus different from the SOS repressor LexA and related repressors that are self‐cleaving. While the IrrE‐mediated cleavage of DdrO was shown in solution after mixing the purified proteins and when coexpressed in *Escherichia coli*, the in vivo IrrE‐dependent DdrO cleavage in *Deinococcus* was observed only after exposure to radiation, in agreement with the radiation‐induced derepression of the RDR regulon. More recently, essentially the same conclusion for the role of IrrE in DdrO cleavage and gene induction was reached in two independent studies on *D. radiodurans* (Devigne et al., 2015; Wang et al., 2015). Additional results have been claimed for IrrE of *D. radiodurans*, namely that it binds, like DdrO, to the promoter regions of RDR genes (Lu, Chen, Xu, Shah, & Hua, 2012; Wang et al., 2015), although other studies indicated that IrrE does not bind DNA (Ohba, Satoh, Yanagisawa, & Narumi, 2005; Vujicic‐Zagar et al., 2009), and that the protease activity of apo‐IrrE could be restored by manganese but, remarkably, not by zinc ions (Wang et al., 2015). It has also been shown that DdrO is essential for viability (Devigne et al., 2015; Ludanyi et al., 2014; Wang et al., 2015).

Several aspects of the IrrE/DdrO‐controlled response in *D. deserti* have not been elucidated. The zinc dependency for activity of *D. deserti* IrrE, its inability to bind DNA, and the gene composition of the RDR regulon have been predicted from results obtained by structural biology and bioinformatics, but not demonstrated experimentally. In addition, it is unknown how radiation triggers the IrrE‐dependent DdrO cleavage in *Deinococcus* and if this in vivo cleavage occurs when DdrO is bound to DNA or not. The in vitro cleavage only required the two proteins purified from *E. coli* and a hitherto unidentified metal ion. Radiation‐stimulated DdrO cleavage in *Deinococcus* may thus depend on a change in metal availability for IrrE and/or in the ability of IrrE to interact appropriately with DdrO. Unfortunately, the seemingly different results reported for IrrE regarding DNA binding and metal specificity are not helpful for better understanding the mechanism by which radiation triggers DdrO cleavage.

In this work we used in vitro and in vivo experiments to investigate which metal ion(s) can activate DdrO cleavage by *D. deserti* IrrE and if the latter can bind to DNA or DNA‐DdrO complex, and multi‐omics experiments to identify IrrE/DdrO‐regulated genes in *D. deserti*. Our results provide evidence that IrrE is a zinc peptidase and indicate that DdrO cleavage in *Deinococcus* is stimulated by increased availability of zinc ions for IrrE. Interaction of IrrE with DNA or DNA‐DdrO complex was not observed. Transcriptomics and proteomics revealed new RDR regulon members that were not predicted previously. The number of IrrE‐regulated genes/proteins, their induction fold, and protein abundance underscore the crucial role of the RDR regulon in the radiation response. The RDR regulon is largely well conserved in more recently sequenced *Deinococcus* bacteria. Remarkably, however, *ddrA*,* ddrD*, and *pprA* are absent in some radiation‐resistant *Deinococcus* species, arguing for the existence of alternative molecular mechanisms.

## 2 Experimental Procedures

### 2.1 Bacterial strains, plasmids, and growth conditions

The strains and plasmids used in this study are listed in Table 1. *Deinococcus* was grown at 30°C with shaking (150 rpm) in 10‐fold diluted tryptic soy broth (TSB/10) supplemented with trace elements (Vujicic‐Zagar et al., 2009). *Escherichia coli* was grown in lysogeny broth medium (LB) at 37°C unless stated otherwise. Antibiotics were used at the following concentrations for *D. deserti*: streptomycin, 10 μg ml^−1^; kanamycin, 10 μg ml^−1^; and for *E. coli*: kanamycin, 50 μg ml^−1^; ampicillin, 100 μg ml^−1^.

**Table 1:** Bacterial strains and plasmids

Strain or plasmid	Genotype or relevant characteristics	Source or reference
Strain		
*E. coli*		
TOP10	F‐ *mcr*A Δ(*mrr*‐*hsd*RMS‐*mcr*BC) Φ80*lac*ZΔM 15 Δ*lac*X74 *rec*A1 *ara*D139 Δ(*ara*,* leu*)7697 *gal*U *gal*K *rps*L (Str^R^) *end*A1 *nup*G	Invitrogen
BL21 Star (DE3)	F‐ *omp*T *hsd*S_B_ (r_B_‐m_B_‐) *gal dcm rne*131 (DE3)	Invitrogen
BL21 (AI)	F‐ *omp*T *hsd*S_B_ (r_B_‐m_B_‐) *gal dcm araB*::T7RNAP*‐tetA*	Invitrogen
*D. deserti*		
RD19	As wild‐type strain VCD115 but streptomycin‐resistant (Str^R^)	(Vujicic‐Zagar et al., 2009)
RD42	As RD19 but Δ*irrE*Ω*kan*	(Vujicic‐Zagar et al., 2009)
RD62	As RD19 but Δ*ddrO*_C_Ω*kan*	(Ludanyi et al., 2014)
*D. radiodurans*		
DSM 20539	Type strain	Laboratory stock
Plasmid		
p12714	*D. deserti irrE* in pET‐TEV	(Vujicic‐Zagar et al., 2009)
pML9	As p12714 but encoding IrrE‐E83Q	(Ludanyi et al., 2014)
pET22ddrO	*D. deserti ddrO*_*C*_ in pET22b	(Ludanyi et al., 2014)
pET SUMO	Expression vector for *E. coli*, Kan^R^	Invitrogen
pET SUMOddrO	*D. deserti ddrO*_C_ in pET SUMO	This work

### 2.2 Protein expression and purification

Expression and purification of IrrE and IrrE‐E83Q (from pET‐TEV) and DdrO_C_ (from pET22b) and removal of the polyhistidine tag from recombinant IrrE was performed as described previously (Ludanyi et al., 2014). To obtain untagged DdrO_C_, the protein was expressed and purified as a polyhistidine‐SUMO‐DdrO_C_ fusion, followed by removal of the His_6_‐SUMO tag using SUMO protease. For this, *ddrO*_C_ (*Deide_20570*) was amplified from *D. deserti* (for primers, see Table S6) and cloned in pET SUMO, resulting in pET SUMOddrO. For expression of His_6_‐SUMO‐tagged DdrO_C_ *, E. coli* BL21 (AI) cells freshly transformed with pET SUMOddrO were grown overnight to saturation in 10 ml of LB containing kanamycin. This preculture was then diluted in 1 L of LB medium with kanamycin (in a 3‐l flask) and grown with shaking (160 rpm) at 37°C. At OD_600_ of 0.6–0.7, IPTG and L‐arabinose were added at a final concentration of 0.1 mmol/L and 0.2%, respectively, and the cells were then grown at 17°C for 17 hr. The induced cells were harvested by centrifugation (5,000 *g*, 20 min, 4°C). The cell pellets were then resuspended in 20 ml of Buffer 1 (500 mmol/L NaCl, 10 mmol/L Na_2_HPO_4_, 1.8 mmol/L KH_2_PO_4_, pH 7.4), and frozen at −20°C. For purification, cell suspensions containing the recombinant fusion protein were thawed and 100 μl of 10 mg ml^−1^ DNase I [DNase from bovine pancreas in 50 mmol/L MgCl_2_ (Sigma)] and 25 μl of anti‐protease cocktail (Sigma P8849) were added to each tube. All purification steps were done at 4°C. Cells were broken by a cell disruptor (One Shot Model, Constant Cell Disruption Systems) at 2 kbar and the soluble extracts were then recovered after centrifugation at 10,000 *g* for 10 min at 4°C and ultracentrifugation at 150,000 *g* for 45 min at 4°C. The supernatant was injected at a speed of 1 ml min^−1^ onto HisTrapTM HP columns (1 ml) (GE Healthcare), previously equilibrated in Buffer 1 supplemented with 20 mmol/L imidazole. A step gradient of imidazole (20, 120 and 500 mmol/L) was used for elution (20 ml was used for each fraction except for the 500 mmol/L fraction where 3 ml was used). Eluted fractions were analyzed by SDS‐PAGE to verify presence and purity of the protein. The polyHis‐SUMO‐tagged DdrO_C_ fusion protein was found in the 500 mmol/L imidazole fraction. The polyHis‐SUMO tag was removed from the fusion protein by incubation with SUMO protease. The pET28b derivative encoding His‐tagged SUMO protease was kindly provided by Mossessova & Lima (2000). The incubation mixture contained one unit of SUMO protease for 20 μg protein, and proteolysis proceeded overnight at room temperature. Then the cleavage reaction was loaded on a nickel affinity column to separate untagged DdrO_C_ (in the flow‐through fractions) from uncleaved fusion protein and SUMO protease (both bound to the column). Protein concentrations were measured using a NanoVue spectrophotometer. The mass of the purified proteins was verified by electrospray ionization mass spectrometry as described previously (Ludanyi et al., 2014).

### 2.3 Metal specificity of the IrrE‐mediated DdrO_C_ cleavage reaction in vitro

Purified IrrE was first incubated with EDTA to remove divalent metal ion and inactivate the protease activity. For this, 20 μl of a 20 mmol/L stock solution of EDTA was added to 2 ml of 40 μmol/L of IrrE. Next, to remove the EDTA, 700 μl of EDTA‐treated IrrE was dialyzed in 350 ml of buffer (50 mmol/L Tris‐HCl pH 7.4, 0.15 mol/L NaCl) for 2 hr and then overnight in fresh buffer. Then cleavage of purified DdrO_C_ was assayed in vitro by incubating 20 μmol/L DdrO_C_ with 5 μmol/L IrrE in a reaction mixture containing 50 mmol/L Tris‐HCl pH 7.4, 0.15 mol/L NaCl, in presence or absence of one of several metal ions (added from freshly prepared metal chloride stock solutions) in a 20 μl final volume. All the reaction mixtures were incubated at 37°C for 15 min. After quenching by addition of NuPAGE LDS sample buffer (Invitrogen) and heating at 95°C for 10 min, the reaction products were subjected to a Novex NuPAGE 10% Bis‐Tris Gel (Invitrogen). Proteins were visualized by staining with Imperial protein stain (Pierce).

### 2.4 Metal shock‐induced DdrO_C_ cleavage in vivo

Strains were grown to exponential phase (OD_600_ 0.4). Then 250 μmol/L of ZnCl_2_ or other metal chloride was added to the 50‐ml culture, and incubation was continued for 10 min. Preparation of cell extracts, protein separation and immunoblot analysis to analyze DdrO cleavage were performed as described previously (Ludanyi et al., 2014). Protein concentration of cell extracts was determined by the CooAssay Protein Dosage Reagent UPF86420 (Uptima/Interchim).

### 2.5 Electrophoretic mobility shift assay

The promoter region of *dnaK* (*Deide_21970*), *ddrA* (*Deide_09150*) or *ddrD* (*Deide_01160*), or the intergenic region of the divergent *ddrO*_C_ (*Deide_20570*) and *ddrQ* (*Deide_20580*) genes were amplified by PCR (Fig. S1; Table S6). DNA binding reactions were performed for 30 min at room temperature in a volume of 20 μl containing 200 ng of DNA fragments and purified DdrO_C_ and/or IrrE in 10 mmol/L Tris‐HCl pH 7.4, 60 mmol/L NaCl, 1 mmol/L DTT, 5% glycerol. When both IrrE (or IrrE‐E83Q) and DdrO were present, either IrrE and DdrO were preincubated for 15 min prior to the incubation with DNA, or DdrO and DNA were incubated prior to addition of IrrE and another 15 min incubation. After adding 2 μl of 10X Orange Loading Dye, the samples were loaded onto a prerun 5% polyacrylamide gel and run for 2 hr at 70 V in TAE buffer at 4°C. The gel was then incubated for 30 min in ethidium bromide (0.5 μg ml^−1^) and DNA was visualized using UV.

### 2.6 RNA sequencing and analysis

Growth, irradiation, RNA isolation, cDNA library construction, Illumina sequencing and RNA‐Seq analysis for strains RD42 and RD62 were performed as described previously for strain RD19 (de Groot et al., 2014), except that only the cDNA synthesis protocol without Terminator exonuclease treatment was used. Briefly, exponential phase cells were exposed to 0 or 1 kGy gamma radiation at room temperature and then recovered for 30 min, followed by addition of RNAprotect Bacteria Reagent (Qiagen) to stabilize RNA. The available budget allowed RNA sequencing of one sample for each strain and condition. The RNA‐Seq data for RD19 were published previously (de Groot et al., 2014). The data set for RD42 and RD62 has been deposited in National Center for Biotechnology Information's Gene Expression Omnibus and is accessible through GEO Series accession number GSE95658.

### 2.7 Proteomics

A 100 ml‐culture of each strain was grown to exponential phase (OD_600_ 0.3), concentrated 100X, and half of the sample was irradiated on ice to 3 kGy gamma rays (32 Gy min^−1^, ^60^Co source) at the now dismantled radiation facilities CIGAL (CEA Cadarache, France). The other half of the sample was not irradiated but otherwise treated in the same manner. After irradiation, cells were diluted 100X in fresh growth medium and recovered for 1 hr, harvested by centrifugation, and washed twice with 50 mmol/L Tris‐HCl pH 7.4. Cell pellets were rapidly frozen in liquid N_2_ and stored at −80°C. Cells were solubilized in lithium dodecyl sulfate sample loading buffer (Invitrogen) as previously described (Hartmann, Allain, Gaillard, Pible, & Armengaud, 2014) and heated at 99°C for 5 min. The released proteins were subjected to a short SDS‐PAGE and stained with Coomassie Blue Safe stain (Invitrogen). Five protein bands of equal size were excised from each lane from high‐molecular weight to low‐molecular weight. They were then processed for trypsin proteolysis as described (Clair, Roussi, Armengaud, & Duport, 2010) in presence of 0.01% of proteaseMAX surfactant in order to maximize the extraction of peptides. NanoLC‐MS/MS identification of peptides was performed on a LTQ‐Orbitrap XL hybrid mass spectrometer (ThermoFisher) coupled to an UltiMate 3000 LC system (Dionex‐LC Packings) operated as described previously (Clair et al., 2010). MS/MS spectra assignment was achieved after merging the five nanoLC‐MS/MS records for each proteome sample with the MASCOT search engine (version 2.2.04) from Matrix Science with full‐trypsin specificity, a mass tolerance of 10 ppm on the parent ion and 0.5 Da on the MS/MS, static modifications of carboxyamidomethylated Cys (+57.0215), dynamic modifications of oxidized Met (+15.9949), and a possible missed cleavage. Normalized spectral count comparisons have been performed as described, normalizing the sum of spectral counts with the predicted molecular weight of each polypeptide (Christie‐Oleza, Fernandez, Nogales, Bosch, & Armengaud, 2012). Proteome data were obtained for two (non‐irradiated RD19) or three biological replicates (all other strains and conditions).

### 2.8 Bioinformatics

MicroScope (previously named MaGe), a platform for microbial genome annotation and comparative analysis (Vallenet et al., 2013), was used to find gene homologs of radiation‐induced genes in 7 *Deinococcus* genomes, and to extract the nucleotide sequences (from −300 to +60 relative to the translation initiation codon) of these genes. A conserved 17‐bp palindromic motif was searched in these extracted sequences using MEME (Bailey & Elkan, 1994).

## 3 Results

### 3.1 Restoration of in vitro IrrE peptidase activity by Zn^2+^, Mn^2+^, and Fe^2+^

**Figure 1:**
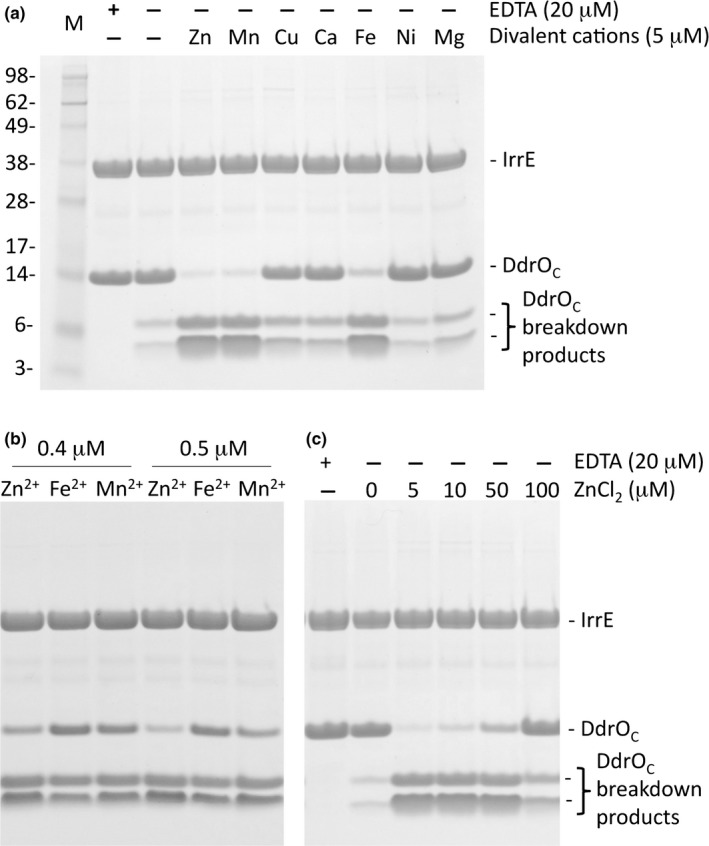
Different metal ions restore peptidase activity of apo‐IrrE in vitro. Purified IrrE was incubated with EDTA to chelate divalent metal ions. After removing EDTA, different metal ions were tested for restoration of peptidase activity of IrrE (5 μmol/L), which was monitored by the cleavage of its substrate DdrO (20 μmol/L). (a) IrrE and metal ions in equimolar concentration. Lane M contains molecular weight marker proteins (masses, in kDa, are indicated). (b) Lower concentration of metal ions compared to IrrE. (c) Excess zinc inhibits IrrE‐mediated DdrO cleavage

We previously demonstrated that the in vitro cleavage of DdrO by IrrE is inhibited in presence of the metal chelator EDTA. Here, we tested several divalent metal ions for their capacity to restore the peptidase activity of apo‐IrrE in vitro. DdrO cleavage was found in the presence of Zn^2+^, Mn^2+^, and Fe^2+^, but not with Cu^2+^, Ca^2+^, Ni^2+^, and Mg^2+^ (Figure 1a). The addition of appropriate metal ions to dialyzed apo‐IrrE is thus sufficient to obtain the protease able to cleave DdrO. The most efficient cleavage was observed with Zn^2+^ when lower amounts of metal ions were added (Figure 1b). We also observed some DdrO cleavage after dialysis to remove the EDTA but without addition of metal ions, most likely because of the presence of a low level of metal contamination. The presence of zinc as contaminant in laboratory buffers and commonly used labware has been described (Kay, 2004). Zn^2+^, Mn^2+^, and Fe^2+^ could also restore peptidase activity when added to the inactive apo form of the zinc metalloprotease thermolysin of *B*. *thermoproteolyticus* (Holmquist & Vallee, 1974). Remarkably, our result with IrrE from *D. deserti* is different from that reported for IrrE from *D. radiodurans*, for which the same metal ions were tested but where only Mn^2+^ was found to restore the in vitro protease activity (Wang et al., 2015). Excess zinc was shown to inhibit the in vitro activity of thermolysin (Holmquist & Vallee, 1974), which was explained by the presence of a second zinc ion that was bound close to the native zinc in thermolysin (Holland, Hausrath, Juers, & Matthews, 1995). We observed that excess zinc also inhibited the peptidase activity of *D. deserti* IrrE (Figure 1c). As for thermolysin, the inhibition of IrrE by excess zinc may result from binding of a second zinc ion in the active site. Indeed, besides the crystal structure of IrrE containing a single zinc ion (Vujicic‐Zagar et al., 2009), another structure with two zinc ions bound in the active site has been previously obtained, after prolonged soaking of apo‐IrrE crystals in a solution containing Zn^2+^, and deposited in the Protein Data Bank (RCSB PDB accession code 3DTK). Together, the data do not support the previously published claim that the peptidase activity of IrrE is strictly dependent on Mn^2+^, but instead support that IrrE is a zinc peptidase.

### 3.2 Zinc shock induces IrrE‐dependent DdrO cleavage in *Deinococcus*

The mechanism by which DdrO cleavage by the constitutively expressed IrrE is stimulated after exposure of *Deinococcus* to radiation is unknown. In vitro, adding zinc ions to apo‐IrrE is sufficient to induce protease activity and DdrO cleavage. The in vivo protease activity of IrrE may similarly depend on the availability of the metal cofactor. In cells, free zinc levels are low because nearly all zinc ions are bound to proteins and other molecules. IrrE is a low‐abundance protein in *D. deserti* (de Groot et al., 2009; Ludanyi et al., 2014), and therefore most of the intracellular zinc may be bound to more abundant proteins resulting in limited availability for IrrE. It has been reported that oxidative stress can result in rapid release of Zn^2+^ from cysteine‐containing zinc sites, resulting in increased levels of free zinc allowing novel interactions between Zn^2+^ and other proteins (Kröncke & Klotz, 2009; Maret, 2006). In *Deinococcus*, such zinc release caused by radiation or desiccation may increase the availability of zinc for IrrE.

**Figure 2:**
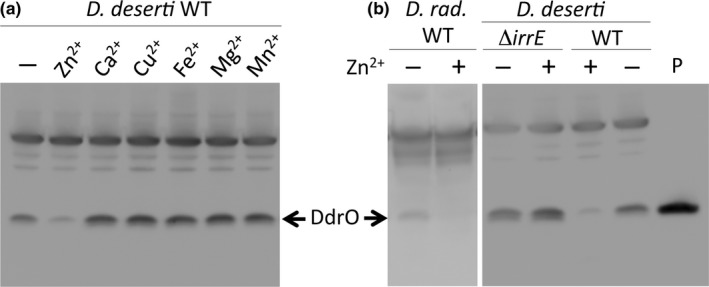
Zinc shock induces IrrE‐dependent DdrO cleavage in vivo. Exponential phase cultures were exposed for 10 min to 250 μmol/L of the indicated metal ions, and analyzed by Western blotting using an antiserum raised against DdrO_C_ of *D. deserti*. Forty μg of cell extract protein was loaded in each lane, except for lane P where 20 ng of purified DdrO_C_ was loaded. (a) *D. deserti* wild‐type strain (WT). (b) *D. radiodurans* wild‐type (*D. rad*. WT) and *D. deserti irrE* mutant (Δ*irrE*) and wild‐type strains

To investigate if increased Zn^2+^ levels may activate the IrrE protease, cultures of *D. deserti* were challenged with zinc. Such a zinc shock was shown to result in a rapid increase in intracellular zinc concentration in different bacteria (Ma et al., 2014; Wang, Hosteen, & Fierke, 2012). *Deinococcus deserti* cells were collected 10 minutes after addition of ZnCl_2_. Western blotting revealed that this zinc shock indeed resulted in cleavage of DdrO in wild‐type cells (Figure 2a and b), but not in the *irrE* deletion mutant (Δ*irrE*) (Figure 2b). Cleavage was also not observed when the cells were equally treated with other metal ions (Figure 2a). Zinc‐induced cleavage also occurred in *D. radiodurans* (Figure 2b). The results support the hypothesis that an increased availability of zinc for IrrE causes induction of the RDR regulon.

Also an increased amount of IrrE may, by competition for Zn^2+^, lead to more Zn^2+^‐containing IrrE. Indeed, overexpression of IrrE and DdrO in *E. coli* showed cleavage of DdrO without applying a stress condition (Ludanyi et al., 2014). Moreover, a transposon insertion upstream of *irrE* in *D. radiodurans* resulted in increased expression of genes of the RDR regulon, probably because of enhanced expression of *irrE* caused by the transposon (Devigne et al., 2015).

### 3.3 Binding of DdrO, but not of IrrE, to RDRM‐containing DNA

**Figure 3:**
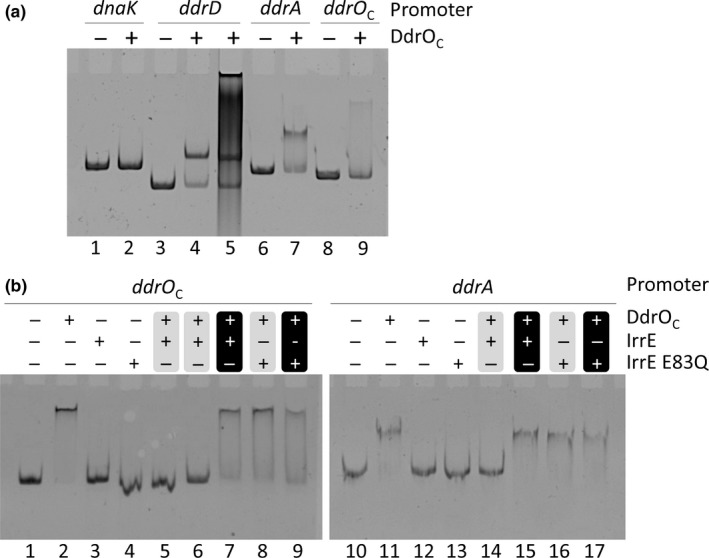
Effect of IrrE on binding of DdrO to RDRM‐containing promoter regions. (a) DdrO (0.8 μmol/L) was incubated with DNA fragments containing the promoter of *dnaK*,* ddrA* or *ddrD*, or the intergenic region of the divergent *ddr* *O*_C_ and *Deide_20580* genes that are both radiation‐induced (DNA fragments at 51, 64, 57 and 57 nmol/L, respectively). The sample in lane 5 also contained 1 μg of Poly(dI‐dC). (b) Electrophoretic mobility shift assays (EMSA) with DNA fragments containing *ddr* *O*_C_‐*Deide_20580* intergenic region or *ddrA* promoter region in presence of IrrE. When both IrrE (or IrrE‐E83Q) and DdrO were present (1.7 μmol/L each), either IrrE and DdrO were pre‐incubated at 37°C (lane 5) or room temperature (lanes 6, 8, 14 and 16) prior to addition of DNA (symbols + and ‐ in grey background), or DdrO and DNA were incubated prior to addition of and incubation with IrrE (lanes 7, 9, 15 and 17; white symbols in black background)

In earlier electrophoretic mobility shift assays (EMSA) we did not observe binding of *D. deserti* DdrO (with a C‐terminal His_6_‐tag) to short biotin‐labeled RDRM‐containing DNA fragments, probably because the experimental conditions were not optimal. However, we did demonstrate repression of an RDRM‐containing promoter by DdrO in *E. coli* (Ludanyi et al., 2014). After changing the experimental conditions for EMSA, including the use of DdrO without a purification tag, binding of DdrO to RDR promoter‐containing DNA fragments was now observed (Figure 3a and S1). Recently, such binding was also shown for DdrO from *D. radiodurans* (Wang et al., 2015). A previous study has reported that also IrrE from *D. radiodurans* binds to promoter regions of genes of the RDR regulon (Lu et al., 2012), although other data have indicated that IrrE does not bind DNA (Ohba et al., 2005; Vujicic‐Zagar et al., 2009). Here, we did not observe binding of IrrE from *D. deserti* to RDR promoter‐containing DNA fragments (Figure 3b).

The IrrE‐mediated cleavage removes the last 23 residues from DdrO, which probably abolishes DdrO dimerization and stable DNA binding (Ludanyi et al., 2014). Indeed, a DNA mobility shift was no longer observed when DdrO was incubated with IrrE prior to addition of DNA (Figure 3b, lanes 5, 6 and 14). This result was dependent on the protease activity of IrrE because it was not found when DdrO was preincubated with IrrE containing the active site mutation E83Q (Figure 3b, lanes 8 and 16). Interestingly, the same band shift was still found when the DdrO‐DNA complex was allowed to form before incubation with IrrE, indicating that IrrE does not efficiently cleave DNA‐bound DdrO and that IrrE (or IrrE E83Q) does not bind to DdrO‐bound DNA (Figure 3b, lanes 7, 9, 15 and 17).

### 3.4 Transcriptomics reveals new IrrE/DdrO‐regulated genes

The genes that are part of the IrrE/DdrO‐controlled RDR regulon in *D. deserti* have been predicted by bioinformatics (i.e., by the identification of an RDRM site) (de Groot et al., 2009, 2014). To experimentally confirm or identify IrrE/DdrO‐regulated genes, we analyzed and compared *D. deserti* wild‐type strain RD19, the *irrE* deletion mutant RD42 (Δ*irrE*), and the *ddrO*_C_ deletion mutant RD62 (Δ*ddrO*_C_) by transcriptomics using RNA sequencing. It should be noted that *D. deserti* contains two DdrO homologs, DdrO_C_ and DdrO_P3_ encoded by the chromosome and plasmid P3, respectively. These proteins share 84% identity and both are cleaved by IrrE in radiation‐exposed cells (Ludanyi et al., 2014). Single *ddrO* deletion mutants could be obtained, but not a strain in which both *ddrO* genes are deleted, indicating that *ddrO* is essential for viability (Ludanyi et al., 2014). However, unlike the *ddrO*_P3_ mutant for which no phenotype was observed, cells of the *ddrO*_C_ mutant showed filamentation and reduced colony‐forming efficiency (L. Blanchard and A. de Groot, unpublished observations), in line with results that showed that DdrO_C_ is the major DdrO protein in *D. deserti* in terms of abundance (Ludanyi et al., 2014). The phenotype of the *ddrO*_C_ mutant might be caused by partial derepression of DdrO‐controlled genes, and therefore this strain was included in the experiments.

Each strain was analyzed after growth under standard condition (non‐irradiated, NI) and after exposure to gamma radiation (IR). RNA sequencing data were thus obtained for six samples: NI and IR for the wild‐type, Δ*irrE* and Δ*ddrO*_C_ strains. As radiation‐induced cleavage of repressor DdrO does not occur in the Δ*irrE* strain (Ludanyi et al., 2014), repression of the IrrE/DdrO‐regulated genes is expected in this strain both before and after irradiation. Higher expression of IrrE/DdrO‐regulated genes is expected in irradiated wild‐type and Δ*ddrO*_C_ strains compared with non‐irradiated wild‐type and Δ*irrE* strains (four comparisons IRvsNI) *and* compared with the irradiated Δ*irrE* strain RD42 (two comparisons IRvs42IR). In addition, if the lower amount of repressor DdrO in the Δ*ddrO*_C_ strain (only DdrO_P3_ is present) indeed causes partial derepression of the target genes, somewhat higher expression of RDR regulon genes may be observed in the non‐irradiated Δ*ddrO*_C_ strain RD62 compared with non‐irradiated wild‐type and Δ*irrE* strains (two comparisons 62NIvsNI).

Read counts and results of the differential expression analyses for all genes are presented in Tables S1 and S2, respectively. IrrE‐dependent radiation‐induced expression was observed for the predicted RDR regulon members, except for the non‐induced *tkt* (transketolase, Deide_00600), and most showed partial derepression in the non‐irradiated *ddrO*_C_ mutant, confirming their predicted regulation by IrrE/DdrO (Table S2). Table 2 shows the 35 most highly radiation‐induced genes. Thirty of these genes were found induced in an IrrE‐dependent manner with a more than 2.5‐fold higher expression in irradiated wild‐type and Δ*ddrO*_C_ strains compared with irradiated Δ*irrE* (column IRvs42IR in Table 2). For several genes these fold changes for IRvs42IR were lower than for IRvsNI because their radiation‐induced expression in Δ*irrE* was reduced but not entirely eliminated (e.g., *gyrB* and *ssb*), perhaps because their induction involves (an) additional regulatory protein(s) besides IrrE/DdrO. An RDRM site has been previously found upstream of most of the highly induced IrrE‐regulated genes (or upstream of the operons *cinA*‐*ligT*‐*recA*_C_, *Deide_18730* to *Deide_18690*,* ssb*‐*rpsR*‐*rplI* and *ddrA*‐*Deide_09148*), but not upstream of *Deide_01090*,* Deide_01100*,* Deide_18350*,* Deide_09130*,* Deide_11446* and *Deide_21600* (Table 2). *Deide_09130* is located 122 nucleotides downstream of *Deide_09148*. Further analysis of this region revealed only few cDNA reads mapped to *Deide_09130* compared to *ddrA*‐*Deide_09148*, and indicated a potential transcription start site (TSS) for *Deide_09130* at the first nucleotide of its ATG start codon (leaderless transcript). Transcription from this TSS was not radiation‐induced. These data suggest that *Deide_09130* is not part of the operon with *ddrA‐Deide_09148* and that its apparent induction is caused by read‐through from the highly induced *ddrA* promoter. Similarly, the induction of *Deide_01100* might be caused by read‐through from the *Deide_01090* promoter. Only few reads were mapped to *Deide_01100* compared to *Deide_01090*. However, as no TSS for *Deide_01100* was found, this gene might be in operon with *Deide_01090*, although these genes are separated by 237 base pairs.

**Table 2:** Most highly radiation‐induced genes in *Deinococcus deserti*

IRvsNI^a^	IRvs42IR^b^	Gene^c^	Product	RDRM^d^
**>10**	**>10**	***Deide_01090***	DinB family protein	
		*Deide_01160*	DNA damage response protein DdrD	+
		***Deide_02990***	DNA damage response protein DdrB	+
		***Deide_04721***	Conserved protein	+
		*Deide_09148*	Putative protein	(+)
		***Deide_09150***	DNA damage response protein DdrA	+
		***Deide_18350***	Holliday junction DNA helicase RuvB	
		*Deide_18730*	SWIM zinc finger domain protein	+
		***Deide_20570***	XRE family transcriptional regulator DdrO_C_	+
		***Deide_20580***	Conserved protein	+
		***Deide_23280***	DNA damage response protein DdrC	+
		***Deide_2p01380***	DNA repair protein PprA	+
		*Deide_3p00210*	RecA_P3_	+
		***Deide_3p02170***	XRE family transcriptional regulator DdrO_P3_	+
	**>5**	*Deide_01100*	DinB family protein	
		***Deide_19440***	2′‐5′ RNA ligase (LigT)	(+)
		***Deide_19450***	RecA_C_	(+)
		*Deide_1p01260*	RecA_P1_	+
	**>2.5**	***Deide_15490***	DNA gyrase, subunit B (GyrB)	+
	**not >2.5**	***Deide_00110***	30S ribosomal protein S18 (RpsR)	(+)
**>5**	**>5**	*Deide_02842*	Type II restriction enzyme	+
		*Deide_09130*	Trans‐aconitate 2‐methyltransferase	
		*Deide_11320*	DNA helicase RecQ	+
		*Deide_18690*	VWA domain‐containing CoxE‐like protein	(+)
		*Deide_18710*	Conserved protein	(+)
		*Deide_18720*	Conserved protein	(+)
		*Deide_19430*	CinA‐like protein	+
	**>2.5**	***Deide_00120***	Single‐stranded DNA‐binding protein (SSB)	+
		***Deide_03120***	Excinuclease ABC subunit B (UvrB)	+
		*Deide_11446*	Putative protein	
		*Deide_21600*	RtcB family protein	
	**not >2.5**	***Deide_00100***	50S ribosomal protein L9 (RplI)	(+)
		*Deide_08010*	ABC transporter permease	
		*Deide_20140*	Putative N‐acetyltransferase	
		*Deide_1p01870*	Repressor LexA_P1_	

a Fold change in irradiated wild‐type *and* Δ*ddrO*_C_ versus non‐irradiated wild‐type *and* Δ*irrE*.

b Fold change in irradiated wild‐type *and* Δ*ddrO*_C_ versus irradiated Δ*irrE* (RD42).

c Bold face indicates a fold change of at least 2.5 in non‐irradiated Δ*ddrO*_C_ versus non‐irradiated wild‐type *and* Δ*irrE*.

d RDRM identified in *previous* studies. +, upstream of the indicated gene; (+), upstream of the operon containing the indicated gene.

The *ddrO*_C_ gene is highly induced in irradiated wild‐type strain. Obviously, *ddrO*_C_ is not expressed in the Δ*ddrO*_C_ strain, because the entire *ddrO*_C_ and 43 bp upstream of this gene are deleted in this strain. However, the TSS of *ddrO*_C_ at position ‐131 of the translation initiation codon is still present in Δ*ddrO*_C_. Differential expression analysis using the number of reads that begin at this TSS showed a more than 25‐fold higher expression in irradiated and non‐irradiated Δ*ddrO*_C_ compared with non‐irradiated wild‐type and Δ*irrE* and with irradiated Δ*irrE* (Table S3). Such expression analysis at the TSS (Tables S3 and S4) was also performed for all other previously reported TSSs (de Groot et al., 2014). This revealed radiation‐induced IrrE‐dependent expression at only one of the two TSSs for *gyrA* (induced at the TSS at ‐83 but not at the TSS at ‐139) and for the *cinA*‐*ligT*‐*recA*_C_ operon (induced at the TSS at ‐9 but not at the TSS at ‐64), correlating with the position of the repressor‐binding motif RDRM. Highly induced IrrE‐dependent expression was also observed at a TSS that is located at ‐181 upstream of *tkt* (*Deide_00600*) within a previously identified RDRM (Fig. S2). However, no radiation‐induced expression was observed for *tkt* itself, which has a TSS located at the first nucleotide of the translation initiation codon (leaderless transcript). This could mean that the TSS at ‐181 of may not correspond to a second TSS for *tkt* but that it may be the TSS for a novel short transcript. No match of this transcript was detected with sequences present in the Rfam database of RNA families. Further work is necessary to characterize this radiation‐induced transcript starting at ‐181 of *tkt*.

The results highlight the crucial role of IrrE in the radiation response in *D. deserti* with respect to both the number of IrrE‐regulated genes and their induction fold, and also indicate additional IrrE‐regulated genes for which an RDRM site was not found in previous bioinformatics studies.

### 3.5 IrrE‐regulated proteins and their abundance

Transcriptome data do not necessarily correlate with protein abundance levels. We therefore also analyzed the *D. deserti* wild‐type and the *irrE* and *ddrO*_C_ mutant strains with a shotgun proteomics procedure, allowing proteome comparison of the different samples and also semiquantitation of the detected proteins by spectral counting. The strains were analyzed after growth under standard condition (non‐irradiated, NI) and after exposure to gamma radiation (IR). A total of 237,894 MS/MS spectra could be assigned when merging all the proteomics data to 7,613 different peptide sequences after querying the database containing the annotated proteins of *D*. *deserti*. In total, 906 proteins were validated with at least two different peptides and compared in terms of abundances (Table S5).

Table 3 presents the 23 proteins found most highly upregulated following irradiation (*p *<* *.05). Thirteen of these proteins were induced in an IrrE‐dependent manner (Tfold > 1.5 in the comparison irradiated wild‐type versus irradiated Δ*irrE*), including ten proteins encoded by genes of the predicted RDR regulon, of which eight proteins were also found derepressed in non‐irradiated Δ*ddrO*_C_ compared with non‐irradiated wild‐type (Tfold > 1.5). Compared to previous proteome studies, the RDR regulon member Deide_20580, hitherto annotated as hypothetical protein, was detected here for the first time, thus validating its existence as a protein. Deide_20580 is encoded by the gene adjacent and divergently oriented to *ddrO*_C_, but the function of this 83‐amino‐acid‐residue protein is unknown. Normalized spectral abundance factors (NSAF) were calculated to estimate the relative quantities of the proteins (Table S5 and Table 3), indicating that RecA (both RecA_C_ and RecA_P_), DdrB, DdrD, GyrA, GyrB, PprA and SSB are the most abundant highly radiation‐induced proteins. It should be noted that the NSAF and Tfold values for RecA_C_ and RecA_P_ in the tables were calculated by taking into account only the spectra corresponding to peptides that distinguish between RecA_C_ and RecA_P_. Many spectra for RecA peptide sequences that are common to RecA_C_ and RecA_P_ isoforms were also obtained, and their quantities in the different samples are in line with the IrrE/DdrO‐regulated expression of RecA (data not shown). Considering all RecA peptides, the relative abundance of total RecA protein is about twice as high as indicated in the tables.

**Table 3:** Most highly radiation‐induced proteins identified by proteome shotgun analysis

19IR vs 19NI^a^	19IR vs 42IR^b^	NSAF			Protein^f^	RDRM^g^
		All^c^	NI^d^	IR^e^		
**>5**	**>5**	5.4	0.0	4.9	Deide_01090 (DinB family protein)	
		20.5	0.1	15.9	**Deide_01160** (DdrD)	+
		0.8	0.3	0.4	Deide_06140 (ABC transporter)	
		5.4	0.0	5.4	Deide_09150 (DdrA)	+
		0.7	0.1	0.6	Deide_19830 (ABC transporter)	
		9.4	0.0	7.5	**Deide_1p01260/Deide_3p00210** (RecA_P_)^h^	+
		2.8	0.0	2.8	Deide_20580 (conserved protein)	+
	**>2.5**	24.9	0.5	20.2	**Deide_02990** (DdrB)	+
		9.7	0.5	6.5	**Deide_19450** (RecA_C_)^h^	(+)
	**>1.5**	7.4	0.5	4.9	**Deide_02842** (restriction enzyme)	+
		16.9	0.7	11.4	**Deide_2p01380** (PprA)	+
	**Not >1.5 with** ***p *** **<** *** *** **.05**	21.9	2.1	10.1	**Deide_00120** (SSB)	+
		0.5	0.0	0.4	Deide_06830 (phage tail sheath protein)	
		3.3	0.4	1.6	Deide_18020 (alanine dehydrogenase)	
		1.1	0.0	0.7	Deide_19040 (GTP‐binding protein HflX)	
		5.6	0.0	3.7	Deide_20140 (N‐acetyltransferase)	
		1.2	0.0	0.8	Deide_23470 (branched‐chain alpha‐keto acid dehydrogenase subunit E2)	
**>2.5**	**>1.5**	13.7	2.1	7.2	**Deide_12520** (GyrA)	+
		22.6	2.6	13.2	**Deide_15490** (GyrB)	+
	**Not >1.5 with** ***p *** **<** *** *** **.05**	3.0	0.2	1.9	Deide_01730 (divalent‐cation tolerance protein)	
		1.3	0.2	0.7	Deide_12320 (conserved protein)	
		5.6	0.6	2.3	Deide_22200 (GTPase Obg)	
		2.7	0.5	1.2	**Deide_3p01130** (ABC transporter)	

a Tfold for comparison irradiated versus non‐irradiated of the wild‐type (RD19) (*p *<* *.05).

b Tfold for comparison irradiated wild‐type versus irradiated Δ*irrE* (RD42) (*p *<* *.05).

c Accumulated for all samples.

d For all samples of non‐irradiated wild‐type and Δ*irrE*.

e For all samples of irradiated wild‐type and Δ*ddrO*_C_.

f Bold face indicates Tfold > 1.5 (*p *<* *.05) for comparison non‐irradiated Δ*ddrO*_C_ versus non‐irradiated wild‐type.

g RDRM identified in *previous* studies. +, upstream of the indicated gene; (+), upstream of the operon containing the indicated gene.

h Data for RecA_C_ and RecA_P_ calculated by taking into account only the peptides that distinguish between RecA_C_ and RecA_P_.

Based on the NSAF values, members of the RDR regulon were found most abundant among the identified highly radiation‐induced proteins (Table 3), showing the importance of this regulon also at the protein level. Two additional proteins were also found with relative high quantity after irradiation, the DinB‐family protein Deide_01090 and the putative N‐acetyltransferase Deide_20140, with Deide_01090 being upregulated in an IrrE‐dependent manner.

### 3.6 New RDR members in *Deinococcus deserti*

The RNA sequencing and proteome shotgun analysis confirmed IrrE‐regulated expression of predicted RDR regulon genes, and also revealed IrrE‐dependent radiation‐induced expression of genes for which an RDRM site was not found in previous studies. To investigate this further, the nucleotide sequences upstream of the induced genes were analyzed for the presence of conserved motifs using the online tool MEME (Bailey & Elkan, 1994). Besides confirming the presence of the RDRM sites that were previously identified with another tool (de Groot et al., 2009), additional RDRM sites were found upstream of *Deide_01090* (DinB family protein) and *Deide_18350* (Holliday junction DNA helicase RuvB). Moreover, these novel RDRM sites (17‐bp motif starting at ‐38 and ‐35 relative to the translation initiation codon of *Deide_01090* and *Deide_18350*, respectively) overlap with the promoter sequences (TSS at ‐25 and +1 for *Deide_01090* and *Deide_18350*, respectively), correlating with DdrO functioning as a repressor also for these genes. The IrrE‐dependent regulation and RDRM sites thus identified *Deide_01090* and *Deide_18350* as new members of the RDR regulon in *D. deserti*. RDRM sites were not found upstream of *Deide_11446* and *Deide_21600*, indicating that these genes might not be regulated directly by DdrO or that their upstream region might contain a weakly conserved RDRM that was not detected in these searches.

### 3.7 Conservation of the RDR regulon in *Deinococcus* species

The IrrE and DdrO proteins are highly conserved in different *Deinococcus* species, and therefore it can be assumed that the radiation response mechanism involving cleavage of DdrO by IrrE is a general trait in these bacteria (Ludanyi et al., 2014). However, comparative analysis revealed that the predicted RDR regulons of *D. deserti*,* D. radiodurans* and *D. geothermalis* are not composed of entirely the same set of genes (de Groot et al., 2009; Makarova & Daly, 2010). For example, a homolog of the RDR regulon member *Deide_04721* of *D. deserti*, encoding a small protein with two pairs of conserved CXXC residues, is present in *D. geothermalis* but not in *D. radiodurans*. To characterize the conservation of the RDR regulon, homologs of radiation‐induced RDR regulon genes from *D. deserti* and *D. radiodurans* were searched in more recently released complete and assembled *Deinococcus* genomes, and the nucleotide sequences upstream of these homologs were analyzed for the presence of the RDRM using MEME. The results are presented in Table 4. Remarkably, homologs of the highly radiation‐induced genes *ddrA*,* ddrD* and *pprA* are not present in each radiation‐resistant *Deinococcus* species. *D. proteolyticus* lacks *ddrA* and *pprA*, and *D. peraridilitoris* lacks *ddrD*. Another radiation‐induced gene, *ddrF*, was found only in *D. radiodurans*. Furthermore, *D. proteolyticus* lacks *cinA* (competence/damage‐inducible protein CinA) and possesses a probable operon with *recA* preceded only by *ligT* (2′‐5′ RNA ligase) instead of the *cinA*‐*ligT*‐*recA* operon present in others. Interestingly, a second *recA* is present in *D. peraridilitoris*, similar to *D. deserti* that has two additional *recA* genes. Homologs of the *D. deserti* RDR regulon genes *Deide_20580*,* Deide_01090*,* Deide_04721* and the five‐gene operon *Deide_18730* to *Deide_18690*, all encoding proteins of unknown function, are present in several other *Deinococcus* species.

**Table 4:** RDR regulon in *Deinococcus* species

*D. deserti* locus tag(s)	Gene(s)	Ddes		Drad		Dgeo		Dgob		Dmar		Dper		Dpro	
		G	M	G	M	G	M	G	M	G	M	G	M	G	M
*Deide_20570*	*ddrO*	+	+	+	+	+	+	+	+	+	+	+	+	+	+
*Deide_20580*	*ddrQ*	+	+			+	+	+	+	+	+	+	+	+	+
*Deide_3p02170*	extra *ddrO*	+	+					+				+			
*Deide_09150*	*ddrA*	+	+	+	+	+	+	+	+	+	+	+	+		
*Deide_02990*	*ddrB*	+	+	+	+	+	+	+	+	+	+	+	+	+	+
*Deide_23280*	*ddrC*	+	+	+	+	+	+	+	+	+	+	+	+	+	+
*Deide_01160*	*ddrD*	+	+	+	+	+	+	+	+	+	+			+	+
*‐*	*ddrF*			+	+										
*Deide_2p01380*	*pprA*	+	+	+	+	+	+	+	+	+	+	+	+		
*Deide_19430* a	*recA* operon^b^	+	+	+	+	+	+	+	+	+	+	+	+	+	+
*Deide_1p01260* c	extra *recA*	+	+									+	+		
*Deide_00120*	*ssb* d	+	+	+	+	+	+	+	+	+	+	+	+	+	+
*Deide_12520*	*gyrA*	+	+	+	+	+	+	+	+	+	+	+	+	+	+
*Deide_15490*	*gyrB*	+	+	+	+	+	+	+	+	+	+	+	+	+	+
*Deide_16210*	*recD*	+	+	+	+	+		+	+	+	+	+	+	+	+
*Deide_11320*	*recQ*	+	+	+	+	+	+	+	+	+		+	+	+	
*Deide_18350*	*ruvB*	+	+	+	+	+	+	+	+	+	+	+	+	+	+
*Deide_12760*	*uvrA*	+	+	+	+	+	+	+	+	+	+	+	+	+	+
*Deide_03120*	*uvrB*	+	+	+	+	+	+	+	+	+	+	+	+	+	+
*Deide_12100*	*uvrD*	+	+	+	+	+	+	+	+	+	+	+		+	
*Deide_00600*	*tkt* e	+	+	+	+	+	+	+	+	+		+		+	
*Deide_01090*	*ddrR*	+	+	+	+			+	+	+		+		+	
*Deide_02842*	*Deide_02842*	+	+												
*Deide_04721*	*ddrS*	+	+			+	+			+	+	+	+	+	+
*Deide_18730* f	*ddrTUVWX*	+	+					+	+	+	+	+	+		

The + indicates the presence of the gene (G) or a detected RDR motif (M) in its upstream region. Black indicates absence of the gene or that an RDRM was not found. Ddes, *D. deserti*; Drad, *D. radiodurans*; Dgeo, *D. geothermalis*; Dgob, *D. gobiensis*; Dmar, *D. maricopensis*; Dper, *D. peraridilitoris*; Dpro, *D. proteolyticus*.

a *Deide_19430*‐*Deide_19440*‐*Deide_19450* operon.

b *ligT*‐*recA* in *D. proteolyticus*,* cinA*‐*ligT*‐*recA* in others.

c And *Deide_3p00210*.

d *ssb‐rpsR‐rplI* operon.

e In *D. deserti*, radiation‐induced expression was found for a transcript starting at –181 of *tkt*, but not for the leaderless *tkt* mRNA.

f Operon *Deide_18730* to *Deide_18690*.

The conserved motif search revealed at least one RDRM site upstream of each homolog, when present, of the following genes in the analyzed *Deinococcus* species: *ddrA*,* ddrB*,* ddrC*,* ddrD*,* ddrO*,* Deide_20580*,* pprA*,* cinA* (of the *cinA*‐*ligT*‐*recA* operon, but upstream of *ligT*‐*recA* in *D. proteolyticus*), extra *recA*,* ssb*,* gyrA*,* gyrB*,* uvrA*,* uvrB*,* ruvB*,* Deide_04721*,* Deide_18730*. Interestingly, two RDRM sites were found upstream of each *ddrA*, including *D. geothermalis ddrA* for which an RDRM was not previously reported (Makarova & Daly, 2010; Makarova et al., 2007). Moreover, the positions of both motifs relative to the *ddrA* coding sequence are identical in these species (Fig. S3). An RDRM site was detected upstream of some but not all homologs of *recD*,* recQ*,* uvrD*,* tkt*, and *Deide_01090,* indicating that these genes may not be part of the RDR regulon in each species or that a potential RDRM was not detected because of low sequence conservation. Concerning the two new RDR regulon members that were identified in *D. deserti* in this study, an RDRM site was previously found in *D. radiodurans* and *D. geothermalis* for *ruvB* but not for a homolog of *Deide_01090* (DinB family protein) (Makarova et al., 2007). Here, the motif search indicated an RDRM upstream of the *Deide_01090* homologs *DR_0053* in *D. radiodurans* and *DGo_PA0274* in *D. gobiensis*. Like Deide_01090, DR_0053 was found highly radiation‐induced at the RNA and protein level, and the detected RDRM starting at ‐30 of the start codon overlaps with the reported TSS of the radiation‐induced *DR_0053* transcript (Appukuttan et al., 2015). Because homologs of *Deide_20580*,* Deide_01090*,* Deide_04721* and the five‐gene operon *Deide_18730* to *Deide_18690* are predicted RDR regulon genes also in other *Deinococcus* species, we propose the names *ddrQ*,* ddrR*,* ddrS*, and *ddrTUVWX* for these genes (Table 4). *Deinococcus deserti* DdrQ, DdrR, DdrS, and DdrT to DdrX share respectively 51%–78%, 46%–57%, 54%–69% and 31%–82% identity with the homologs in the other deinococci. Together, the results show that the RDR regulon is largely well conserved in *Deinococcus*, but also reveal interesting differences between the individual species.

## 4 Discussion

Maintenance of genome integrity is essential for cell survival. Radiation‐tolerant *Deinococcus* bacteria are famous for their ability to repair massive DNA damage, including hundreds of double‐strand breaks, generated by high doses of radiation or prolonged desiccation. Several mechanisms were shown to be crucial for extreme radiation‐tolerance and DNA repair in *Deinococcus*. Besides limitation of oxidative protein damage and DNA repair itself, these mechanisms include an efficient DNA damage response to induce expression of genes required for DNA repair and survival. Several years ago, three components were shown or predicted to have an important role in radiation‐ or desiccation‐induced gene expression in *D. radiodurans*,* D. geothermalis*, and *D. deserti*, namely the IrrE and DdrO proteins and the 17‐bp palindromic DNA motif called RDRM present at a variable position upstream of several induced genes, which suggested the presence of a conserved radiation/desiccation response (RDR) regulon (de Groot et al., 2009; Earl et al., 2002; Hua et al., 2003; Makarova et al., 2007; Vujicic‐Zagar et al., 2009). A link between these three components was demonstrated recently: DdrO functions as a repressor by binding to the RDRM that appeared to be located in or very close to the promoter of the radiation‐induced genes, including *ddrO* itself, and the constitutively expressed low‐abundance protein IrrE is a metalloprotease required for proteolytic inactivation of DdrO to derepress the genes, a process that is somehow stimulated after exposure of *Deinococcus* to radiation (de Groot et al., 2014; Devigne et al., 2015; Ludanyi et al., 2014; Wang et al., 2015). The IrrE‐dependent cleavage of DdrO, and thus the upregulated expression of RDR regulon genes, is essential for radiation tolerance. For survival it is also necessary that DdrO cleavage is switched‐off when the stress is alleviated, resulting in reaccumulation of DdrO and re‐repression of RDR genes. Most of the predicted RDR regulon genes are common to *D. radiodurans*,* D. geothermalis* and *D. deserti*, but an RDRM site was also predicted upstream of several *D. deserti* genes for which an RDRM or gene homolog was not found in *D. geothermalis* and/or *D. radiodurans*, which suggested differences in the gene composition of the RDR regulon (de Groot et al., 2009). The aim of the experiments in the present study was to move a step forward in the characterization of the induction and composition of the RDR regulon in *D. deserti* and other *Deinococcus* species.

It was shown previously that the N‐terminal domain of *D. deserti* IrrE exhibits a mono‐zinc metallopeptidase fold with structural similarity to thermolysin, and that briefly soaking apo‐IrrE crystals in a solution with zinc ions resulted in the binding of a zinc ion in the predicted site coordinated by the expected residues His82, His86 and Glu113, which suggested that IrrE could be a zinc‐dependent protease (Vujicic‐Zagar et al., 2009). In this work we showed that adding zinc to apo‐IrrE indeed restored protease activity. As observed for thermolysin and other zinc peptidases (Fukasawa, Hata, Ono, & Hirose, 2011; Holmquist & Vallee, 1974), some other metal ions could also activate the in vitro protease activity of IrrE, but more efficient DdrO cleavage was found with Zn^2+^ than with Mn^2+^ and Fe^2+^. Interestingly, a short exposure of *Deinococcus* to excess Zn^2+^ also resulted in efficient IrrE‐dependent DdrO cleavage in vivo, supporting the possibility that DdrO cleavage is stimulated by an increased availability of Zn^2+^ for IrrE. Such an increase in available zinc ions may explain how radiation triggers DdrO cleavage, because radiation/oxidative stress can result in rapid release of Zn^2+^ from cysteine‐containing zinc sites (Kröncke & Klotz, 2009; Maret, 2006).

We did not observe binding of IrrE to RDRM‐containing DNA fragments or to DdrO‐bound DNA. The results also indicate that IrrE cannot cleave DNA‐bound DdrO. Similarly, it has been shown that self‐cleavage of the SOS repressor LexA can be induced when dissociated from DNA but not when LexA is bound to target DNA (Butala et al., 2011). With cleavage of unbound repressor, the level of repression/de‐repression of regulon genes will correlate with the binding affinity of the repressor for the different target sites.

Concerning metal specificity and DNA binding, the results obtained with *D. deserti* IrrE in this study are different from those published previously for *D. radiodurans* IrrE. The latter was reported to bind DNA and to possess metalloprotease activity strictly dependent on Mn^2+^ (Lu et al., 2012; Wang et al., 2015). These two IrrE proteins share 64% sequence identity. Moreover, expression of *D. deserti* IrrE in a *D. radiodurans irrE* deletion mutant fully restored radiation resistance (Vujicic‐Zagar et al., 2009). It is therefore very likely that these proteins operate in an identical manner and that the different results are caused by differences in the experimental conditions. For the metal specificity experiments, equimolar amount of Zn^2+^ added to apo‐IrrE of *D. deserti* restored DdrO cleavage, whereas this activity was strongly inhibited after adding 100 μmol/L Zn^2+^ to 5 μmol/L apo‐IrrE. Excess zinc also inhibits the in vitro activity of thermolysin (Holmquist & Vallee, 1974). For the experiments with *D. radiodurans* IrrE, 1 to 5 mmol/L Zn^2+^ was added to 8 μmol/L apo‐IrrE (Wang et al., 2015), and this high Zn^2+^/IrrE ratio did probably lead to inhibition of the activity instead of the supposed failure to stimulate DdrO cleavage. Furthermore, several data strongly suggest that the start codon position of *D. radiodurans irrE* has been wrongly predicted and that native IrrE of *D. radiodurans* is 40 residues shorter than annotated (Devigne et al., 2015; Ludanyi et al., 2014). Because the initially annotated translation initiation codon was considered for cloning and expression of *D. radiodurans irrE*, the purified IrrE contained these 40 extra residues, which may have influenced the properties of the protein and therefore the results of experiments. This stresses the importance of experimentally validating N‐termini of proteins as done with *D. deserti* proteome (Baudet et al., 2010; Hartmann & Armengaud, 2014). Taken all together, we conclude that IrrE is a zinc peptidase that does not bind to DNA.

Transcriptomics and proteomics confirmed the IrrE‐dependent upregulation of the *D. deserti* RDR regulon genes predicted previously (de Groot et al., 2009, 2014), including the genes that have no homolog in *D. geothermalis* and/or *D. radiodurans* (e.g., *Deide_04721*,* Deide_18730*). Of the genes common to the predicted RDR regulons of *D. deserti*,* D. radiodurans* and *D. geothermalis* (Makarova & Daly, 2010), only *Deide_00600* (*tkt*, encoding transketolase) was not found induced after irradiation. However, the RDRM upstream of *Deide_00600* appeared to be involved in IrrE‐dependent regulation of a probably noncoding transcript of unknown function. Transcriptomics and proteomics also led to the identification of additional members of the RDR regulon that were not predicted previously by bioinformatics, showing the power of both bioinformatics and experimental data to identify regulon members. The majority of the highly induced genes belong to the RDR regulon, underscoring the importance of the IrrE/DdrO‐regulated response to radiation.

Besides *D. deserti*,* D. radiodurans*, and *D. geothermalis*, the RDR regulon is largely well conserved in other, more recently sequenced *Deinococcus* bacteria, but some unexpected differences were also found. At least 10 genes or operons are common to the predicted RDR regulons in the seven analyzed genomes: *ddrO*,* ddrB*,* ddrC*,* recA* operon, *ssb*,* gyrA*,* gyrB*,* ruvB*,* uvrA*, and *uvrB*. These 10 genes encode DNA repair proteins, repressor DdrO, and uncharacterized protein DdrC. Remarkably, *ddrA*,* ddrD*, and *pprA*, which were shown to contribute to resistance to gamma radiation, UV and/or mitomycin C in *D. radiodurans* (Narumi et al., 2004; Selvam, Duncan, Tanaka, & Battista, 2013; Tanaka et al., 2004) and whose products were found here among the most abundant radiation‐induced proteins, are not present in each radiation‐resistant *Deinococcus* species. In the other species, DdrA, DdrD and PprA are highly conserved with the respective homologs sharing 50 to 80% sequence identity. In particular the *pprA* mutant of *D. radiodurans* was shown to be very sensitive to gamma radiation, and therefore the absence of *pprA* in *D. proteolyticus* is intriguing. PprA binds to DNA, interacts with gyrase, and is required for accurate chromosome segregation after exposure of *D. radiodurans* to ionizing radiation (Devigne et al., 2016; Kota, Charaka, Ringgaard, Waldor, & Misra, 2014; Narumi et al., 2004). DdrA binds to single‐stranded DNA and is proposed to be part of a DNA end‐protection system that helps to preserve genome integrity after exposure to ionizing radiation (Harris et al., 2004). Expression of *D. deserti* DdrA in radiation‐sensitive *D. radiodurans* Δ*ddrA* bacteria fully restored radiation resistance (Gutsche et al., 2008), further supporting a conserved role of DdrA in radiation resistance in the species possessing this protein. DdrD has been found associated with the nucleoid after irradiation, but its function is unknown (Bouthier de la Tour et al., 2013). These data argue against the idea that non‐conserved genes can be ruled‐out for being involved in radiation resistance in *Deinococcus* (Makarova et al., 2007). Instead, they argue for the existence of not only common but also distinct molecular mechanisms required for radiation tolerance in closely related *Deinococcus* species. Like *pprA*,* ddrA* and *ddrD*, also other RDR genes that are not present in each species may have an important role in the response to radiation or other stress (e.g., *ddrQ* and *ddrS*). It will be interesting to unravel the function of the uncharacterized RDR members that are present in all or most *Deinococcus* species (e.g., *ddrC*,* ddrD*,* ddrQ*,* ddrR*).

## Conflict of Interest

None declared.
