# Supplementary material for: Conservation and diversity of the IrrE/DdrO‐controlled radiation response in radiation‐resistant Deinococcus bacteria
Source: Microbiologyopen. 2017 Apr 11;6(4):e00477. doi: 10.1002/mbo3.477 (PMC5552922; doi:10.1002/mbo3.477)
Supplement: Supplementary file 1 [file MBO3-6-na-s001.pdf]

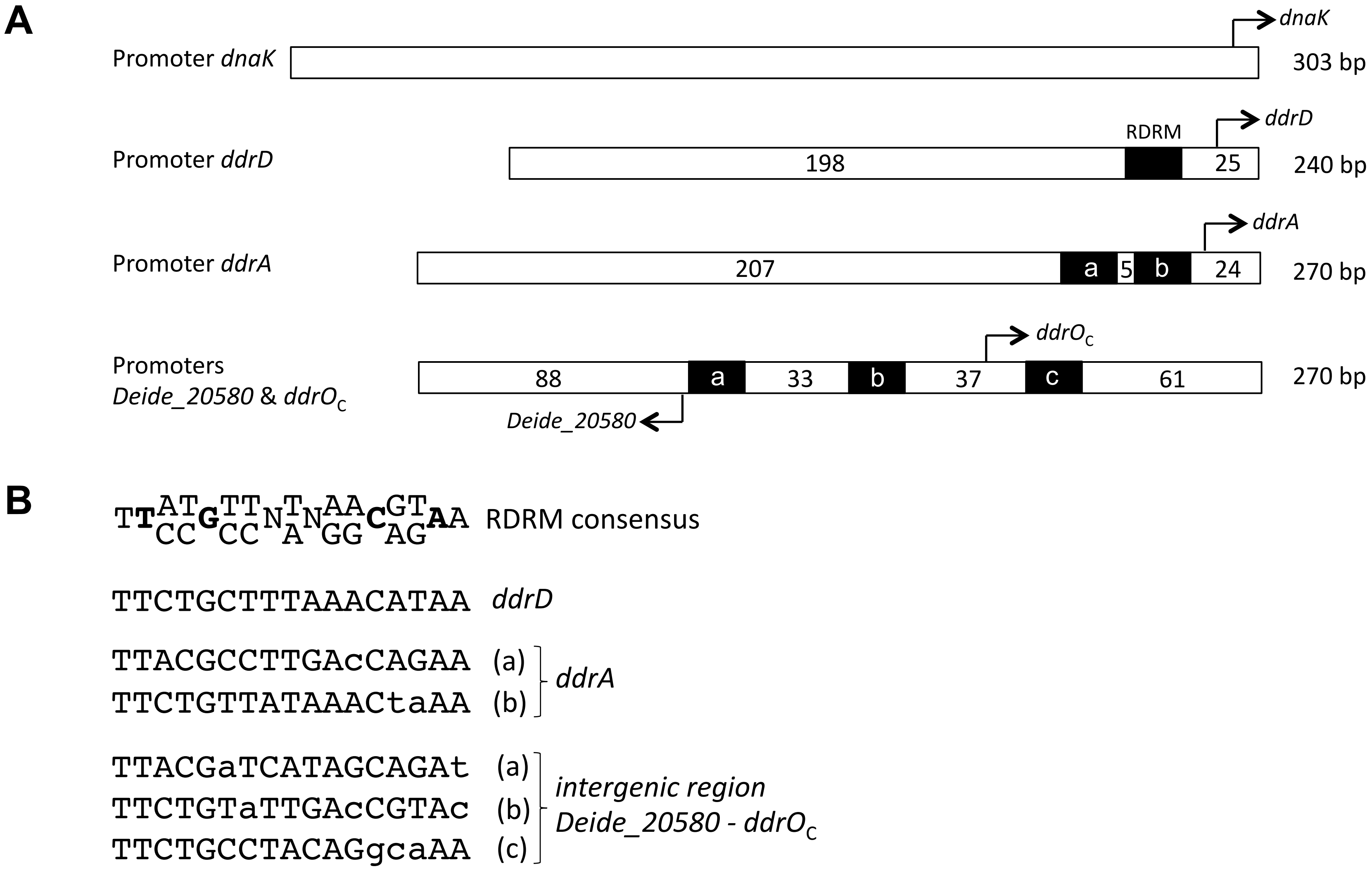

Fig. S1. DNA fragments used in EMSA and their RDRM positions and sequences. (A) Schematic representation of promoter-containing DNA fragments and the positions of the 17-bp RDRM sites (black boxes) and the transcription start sites (arrows). Numbers of base pairs before and after the RDRM sites in these fragments are indicated. (B) Sequences of the RDRM sites (corresponding to the forward strand of the DNA fragments shown in panel A).

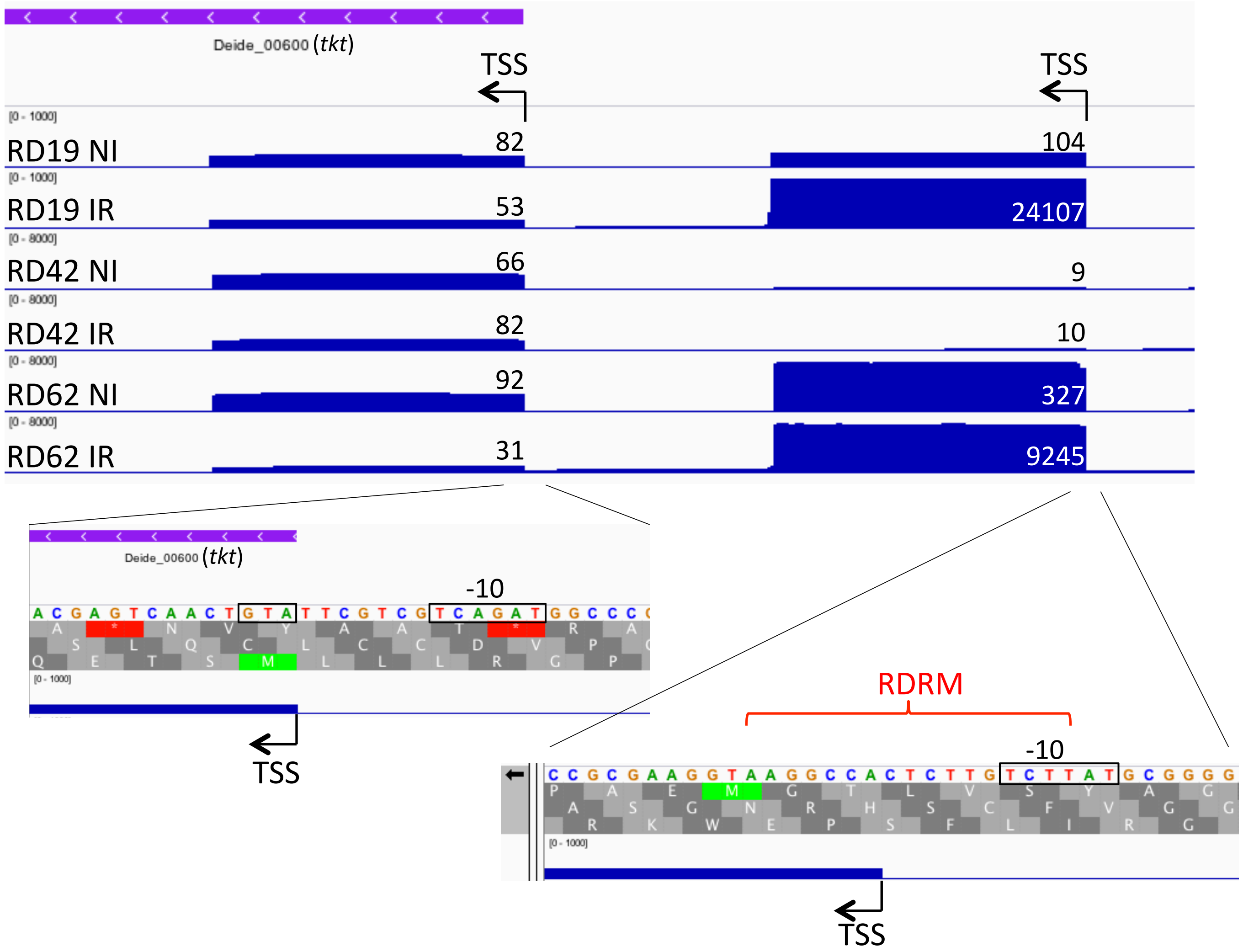

Fig. S2. IrrE-dependent radiation-induced transcription starting at -181 of the transketolase gene. RNA sequencing read coverage in different strains/conditions is indicated in blue. The positions of the transcription start sites (TSS) are indicated, and the numbers below the TSS correspond to the amount of the reads that begin at the TSS (after normalization for the total amount or reads per sample, and per million reads). The images at the bottom are zooms at the TSSs. RD19, wild-type; RD42, Δ*irrE*; RD62, Δ*ddrO*$_C$; NI, non-irradiated; IR, irradiated.

A

Deide_09150
CCTTACTCGGAAGAGGAATTACGCCTTGAcCAGAATTCCATTCTGTTATAAACtaAATCCATG

DR_0423
CCTTAATCAACTGGCGTTTTATGTCTTGAcCGTAATGTTATTCTGTTCTAAACtaAATGCATG

Dgeo_0977
CCCGCTTCTCCCTCCTCATTCTGTCTTGAcCGTAATTGTGTTCTGTTATAAACtaAATCCATG

DGo_CA2046
AAGAGCCGCTCCCTCTCATTCTGTCTTGAcCGTAACTCCGTTACGTTATAGACtaAATACATG

Deima_1833
CTCTCCCCTGCACGCTGGTTACGCATTGAcCAGAATTACATTCTGTTATAAACtaAACATATG

Deipe_3704
GGAAACATATCGACGATATTCTGCCTTGAACAGAACCGTATTCTGTTATAtACtaAATCCATG

B

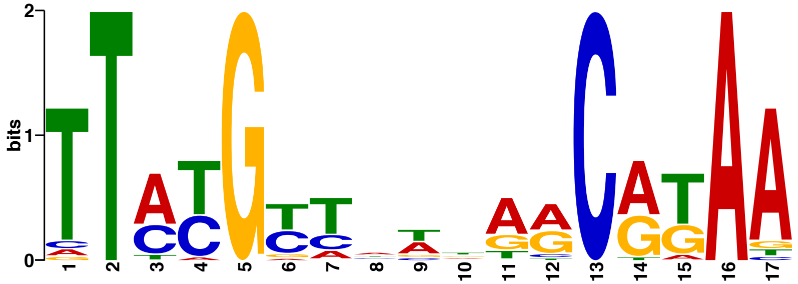

Fig. S3. RDRM sequences upstream of *ddrA* genes and RDRM sequence logo. (A) Two RDRM sites upstream of *ddrA* in different *Deinococcus* species. The 60 nucleotides upstream of the indicated *ddrA* gene are shown (**ATG** corresponds to the translation initiation codon). The RDRM sites are indicated in red. In *D. deserti*, transcription of *ddrA* starts at the first nucleotide of its translation initiation codon (de Groot et al, 2014, Genome Biol Evol 6: 932-948). From the sequence similarities it can be predicted that the *ddrA* mRNAs of the others are also leaderless (note the absence of a Shine-Dalgarno sequence upstream of each start codon). Potential -10 and -35 promoter sequences are underlined. (B) Sequence logo of the RDRM obtained after searching a 17-bp palindromic motif upstream of RDR regulon genes or homologs in seven *Deinococcus* species using MEME (Bailey and Elkan, 1994, Proc Int Conf Intell Syst Mol Biol 2: 28-36). This logo is very similar to the one reported by Makarova *et al* after analysis of *D. radiodurans* and *D. geothermalis* (Makarova et al, 2007, PLoS ONE 2: e955).
