# Supplementary material for: Conservation and diversity of the IrrE/DdrO‐controlled radiation response in radiation‐resistant Deinococcus bacteria
Source: Microbiologyopen. 2017 Apr 11;6(4):e00477. doi: 10.1002/mbo3.477 (PMC5552922; doi:10.1002/mbo3.477)
Supplement: Supplementary file 7 [file MBO3-6-na-s007.pdf]

Table S6. Primers

Primer	Sequence	Amplified fragment
Dd20570sumoFW	ATGAAACTGCACGAACGACTC	*ddrO*$_C$ for cloning in pET SUMO
Dd20570sumoRV	TCAGCTGAGGATGCGTTTC	
prom01160fw	GGGTGAATTCGTAACGGCTGTCAACGGAAAAC	*ddrD* promoter region
prom01160rv	TTTCAGGGTCTAGATATCCGCATTATTATGTTTAAAGCAG	
prom21970fw	GCGTGAATTCCGGGGAGATTCGAGAG	*dnaK* promoter region
prom21970rv	CAGTTCTAGACGTCTGACAGCATAAGGCTAC	
prom20570fw	TATAGGTACCGGTACTCCTTCCTGTCCGAC	Intergenic region with divergently oriented *ddrO*$_C$ and *ddrQ* promoters
prom20570rv	GATACTGCAGCTTTGAGCGACCGGGACT	
prom09150fw	TCTGGGTACCGTGAGCTATGGACCCTC	*ddrA* promoter region
prom09150rv	CTGTACATCGCTCCTGCAGATGGATTTAGTTTATAACAG	
